# Generative Adversarial Networks for Omics Data: An Overview and Future Directions for Livestock Applications

**DOI:** 10.1002/age.70226

**Published:** 2026-10-27

**Authors:** Edgar Caballero Vargas, Tiago Bresolin

**Affiliations:** ^1^ Department of Animal Sciences University of Illinois at Urbana‐Champaign Urbana Illinois USA; ^2^ Center for Digital Agriculture University of Illinois at Urbana‐Champaign Urbana Illinois USA

**Keywords:** data augmentation, generative models, genomics, synthetic data

## Abstract

Generative adversarial networks (GANs) are artificial intelligence models designed to generate novel data that is indistinguishable from real data. Originally developed to generate images to complement existing datasets and create more diverse samples, GAN‐based data can enhance downstream analysis for machine learning and deep learning applications. GAN‐based approaches are also versatile and suitable for augmenting omics data, improving downstream analyses such as genotype prediction, biomarker detection, and protein de novo design. In addition, GANs can preserve and reconstruct genomic regions under selective pressure, increase sample sizes from underrepresented groups, and mitigate class imbalance effects in genomic analyses, transcriptomics, and single‐cell omics. Recent work also demonstrates that GAN‐generated data can support privacy‐preserving data sharing and the design of novel biological sequences and proteins. This literature review presents the foundational concepts of GANs, explores diverse applications across omics fields, and highlights novel opportunities for applying GAN approaches in livestock genomics.

## 1 Introduction

Deep generative models are a class of deep learning methods that learn the underlying probability distribution of complex datasets, enabling the generation of realistic synthetic samples (Hayawi et al. 2024; Rivero‐Garcia et al. 2024). These models have been used to address challenges such as limited sample size, missing data, class imbalance, and privacy constraints through data augmentation, imputation, and privacy‐preserving analysis (Yelmen and Jay 2023). Among generative methods, generative adversarial networks (GANs), introduced by Goodfellow et al. (2014), have become a widely used framework for generating synthetic data (Tripathi et al. 2022). In a GAN, a generator network produces candidate samples from random noise, while a discriminator network learns to distinguish real samples from generated ones (Goodfellow et al. 2014). Through this adversarial training process, the two networks are optimized in opposition until the generator produces samples that closely approximate the target data distribution (Gangwal and Lavecchia 2024). This framework has been applied in several non‐biological areas, achieving state‐of‐the‐art results in natural language processing tasks (Thirumagal and Saruladha 2021), computer vision applications (Olaniyi et al. 2022), video synthesis (Aldausari et al. 2023), and audio generation (Donahue et al. 2019).

Building on their success across these high‐dimensional settings, GANs are increasingly being adopted for omics data, introducing an innovative approach to accelerate research in genomics (Murad et al. 2023; Tripathi et al. 2022). For example, Tamilmani et al. (2022) developed a GAN architecture to synthesize microRNA sequences as biomarkers for cancer detection and showed that augmenting training sets with generated samples can improve classification performance in gene expression analysis. Hazra et al. (2022) applied a GAN‐based approach to generate synthetic cat genomes. The authors reported a mean correlation of 93.7% between real and generated genomes, demonstrating that these models can capture complex biological genomic structure. Similarly, Yelmen et al. (2021) showed that adding GAN‐generated artificial genomes to reference panels increased imputation accuracy by 4.4%, particularly for rare alleles. These examples illustrate that GANs can produce biologically meaningful omics data, laying the groundwork for their application in livestock genomics, where limited data availability and diversity remain key bottlenecks.

Despite these advances, the use of GANs in livestock omics remains largely unexplored. Applying GANs to genomic data from livestock populations under selection raises unique challenges, including preserving biological validity and accurately modeling complex genetic architecture, such as linkage disequilibrium and population structure. In livestock, small effective population sizes, relatedness, and population stratification may influence model performance, making it essential to evaluate whether GANs can generate synthetic omics data that preserve real livestock genomic patterns. Addressing these limitations could open new frontiers in livestock genomics by enabling the simulation of diverse genetic combinations for in silico breeding strategies and, in the long term, the modeling of cellular responses to genetic variation or environmental perturbations (Ghahramani et al. 2018). Thus, this review presents an overview of GAN principles and their applications in omics data and outlines future directions for leveraging GAN‐based approaches to synthesize biologically plausible omics data in livestock. This review does not cover the mathematical foundations or training dynamics of GANs, as prior literature has covered these extensively (Arjovsky et al. 2017; Goodfellow et al. 2014; Gulrajani et al. 2017; Wang 2020).

## 2 Principles of Generative Adversarial Networks

Generative models aim to capture the underlying data distribution and generate synthetic samples that resemble real observations (Yelmen and Jay 2023). Unlike discriminative models, which focus on learning boundary features to distinguish between categories, generative models seek to learn a latent representation of the data distribution (Nayak et al. 2021; Shi et al. 2023). This enables the generation of new synthetic data points that closely approximate real samples (Shi et al. 2023). While several architectures fall under the umbrella of generative modeling, including variational autoencoders (Joo et al. 2020) and Boltzmann machines (GM et al. 2020), generative adversarial networks (GANs) proposed by Goodfellow et al. (2014) have attracted particular attention. Their popularity is largely due to their ability to model complex high‐dimensional data distributions across different data types and to synthesize realistic and diverse artificial data. This section presents the fundamental principles of GANs, which serve as the conceptual basis for the applications discussed in this review paper.

### 2.1 Generative Adversarial Networks Architecture

The GAN framework, introduced by Goodfellow et al. (2014), is grounded in the principle of adversarial training, in which two models compete in a zero‐sum game. This framework is conceptually analogous to biological coevolution, where interacting species continuously adapt in response to one another to maintain a competitive advantage (Papkou et al. 2019; Stewart and Pischedda 2016). For instance, in host–parasite systems, parasites may evolve existing mechanisms to evade host immune defenses, prompting the host to develop novel countermeasures that, in turn, drive further parasite adaptation (Carvalho et al. 2024).

Concepts related to adaptation, competition, and coevolution were incorporated into computational research well before the formal introduction of GANs. Holland (1992), for example, explored adaptive computational systems inspired by biological processes such as evolution and natural selection, while research in artificial life investigated computational systems capable of reproducing characteristics and behaviors observed in living systems through in silico simulation (Banzhaf and McMullin 2012). In the context of machine learning, an early formulation of adversarial competition can be traced to Schmidhuber (1992), who proposed self‐improving systems composed of competing networks. Coevolutionary principles were subsequently incorporated into computational learning frameworks. Li et al. (2013), for example, proposed a framework for learning animal behavior through competitive coevolution, in which behavioral models generated simulated behaviors while a classifier evolved to distinguish real from simulated behaviors. This interaction established a competitive learning process conceptually similar to the generator‐discriminator dynamics later formalized in GANs.

Building on these broader concepts of competitive and adaptive learning, Goodfellow et al. (2014), formalized the GAN framework, in which two neural networks, the generator and the discriminator, are trained simultaneously through an adversarial process. As illustrated in Figure 1, the generator begins with a random vector sampled from a predefined latent distribution, commonly Gaussian or uniform, and learns a mapping from this latent space to the data space. During training, the generator progressively learns to turn these random inputs into realistic synthetic samples that align with the statistical properties of the training data distribution (Alqahtani et al. 2021; Wolterink et al. 2021). Initially, the generator typically produces low‐quality and unrealistic outputs. These generated samples, together with real observations, are fed into the discriminator, which is trained to distinguish real data from samples produced by the generator (Shafahi et al. 2019). Training proceeds by alternately updating the two networks. The discriminator is optimized to improve its ability to distinguish real from generated samples, whereas the generator is optimized using gradients propagated through the discriminator to produce increasingly realistic outputs. In this manner, information from the discriminator provides a learning signal that allows the generator to progressively approximate the empirical distribution of the training data (Goodfellow et al. 2014). Importantly, the discriminator and generator have distinct objective functions and are generally updated separately, although their optimization is inherently interdependent.

**FIGURE 1:**
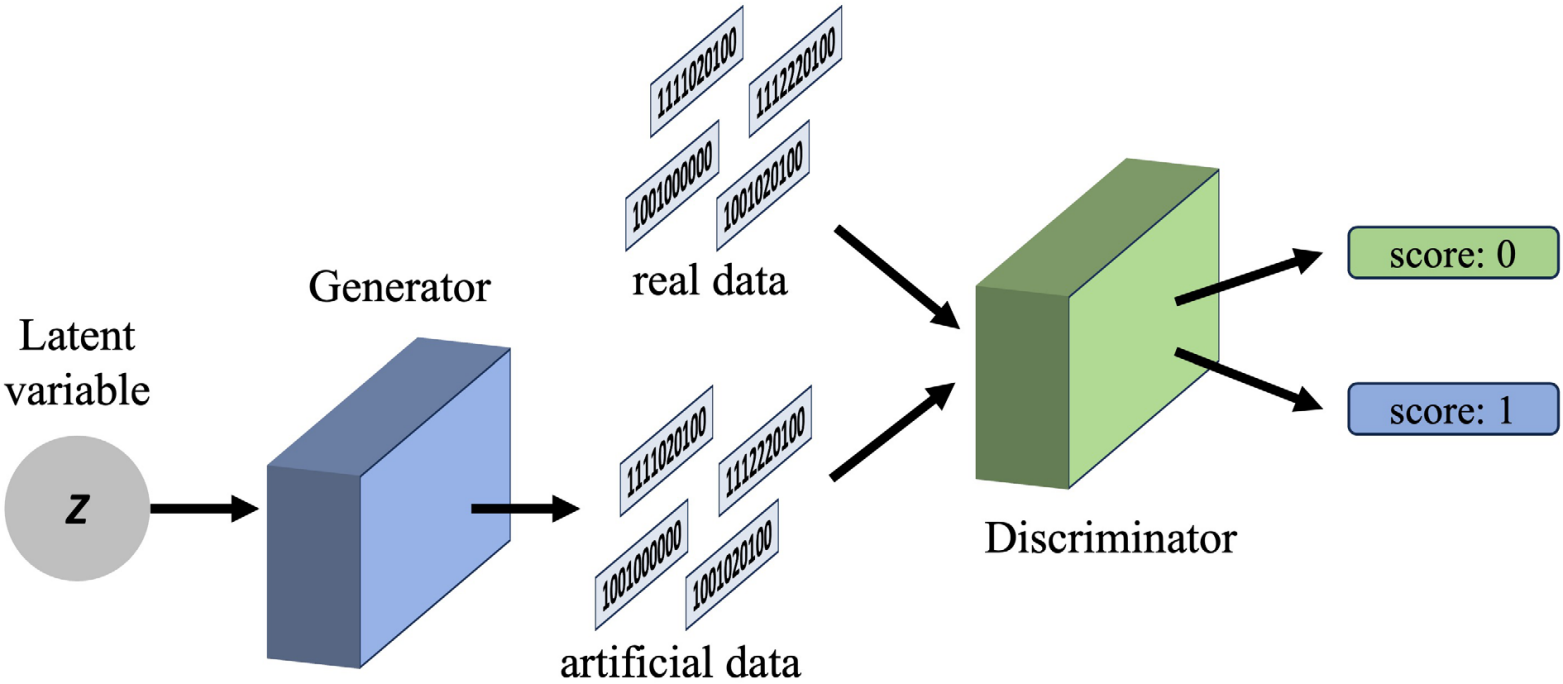
Schematic of a generative adversarial network for synthetic data generation. The generator (blue block) takes random noise from a latent variable *z* (gray circle) and transforms it into artificial samples. These artificial samples are combined with real data and presented to the discriminator (green rectangle), which outputs a score indicating whether each sample is real or generated. During training, the discriminator learns to distinguish real from artificial data, while the generator is updated to produce increasingly realistic samples until the discriminator can no longer distinguish between synthetic and real samples.

This adversarial learning process is commonly formulated as a two‐player minimax game. The discriminator seeks to improve its ability to distinguish real from generated observations, while the generator seeks to produce samples that the discriminator classifies as real. Under the theoretical conditions of the original GAN formulation, the optimal solution is reached when the generated distribution matches the real data distribution. At this equilibrium, an optimal discriminator assigns a probability of 0.5 to both real and generated samples because the two distributions are indistinguishable (Thirumagal and Saruladha 2021; Wenzel 2023). This theoretical state is often described in relation to a Nash equilibrium, although achieving such equilibrium in practical GAN training is considerably more difficult, as discussed in subsequent sections.

The ability of GANs to learn complex high‐dimensional distributions opens new opportunities for applications involving structured biological data, though their role should be distinguished from established genomic simulation approaches. In livestock genomics, probabilistic and mechanistic simulation frameworks, such as QMSim (Sargolzaei and Schenkel 2009) and AlphaSimR (Gaynor et al. 2021), are widely used to generate genomic data under predefined demographic histories, population structures, mating systems, and genetic architectures. These approaches are well established and remain highly effective when the objective is to simulate populations under specified biological scenarios. GAN‐based methods should therefore not be viewed as replacements for conventional genomic simulators, but as complementary data‐driven approaches that address different research objectives. By learning complex patterns directly from empirical genomic data, GANs may be useful for generating synthetic datasets that reproduce features of observed populations, including linkage disequilibrium, haplotype structure, allele‐frequency distributions, and population substructure. Such capabilities may be particularly relevant for applications involving data augmentation, representation of underrepresented populations, privacy‐preserving data sharing, and the development or benchmarking of analytical methods when realistic empirical‐like data are desired. While this section outlines the conceptual architecture of GANs, subsequent sections discuss these applications in greater detail, beginning with the loss functions that govern adversarial training.

### 2.2 Challenges in Training Generative Adversarial Networks

Although GANs have shown potential for generating realistic synthetic data, training them remains a challenge to widespread use. The adversarial dynamics, while powerful, are inherently unstable; the generator and discriminator often fail to synchronize, leading to convergence failures. Additional challenges, such as mode collapse and the absence of universal evaluation metrics, further hinder their real‐world applicability, particularly in genomics, where both biological fidelity and population‐level diversity are non‐negotiable. This section reviews three major categories of challenges: training instability, mode collapse, and evaluation metrics, highlighting their specific impacts on livestock omics data.

#### 2.2.1 Training Instability

The theoretical objective of GAN training is to reach a Nash Equilibrium, where the discriminator no longer distinguishes between real and generated data (Wolterink et al. 2020). In practice, however, achieving this equilibrium is exceptionally difficult because both networks pursue opposing goals: the generator aims to maximize the probability that the discriminator makes a mistake, while the discriminator aims to maximize its accuracy in distinguishing real from synthetic inputs (Salimans et al. 2016). These opposing forces often lead to unstable training, in which neither network converges to an optimal state, resulting in unrealistic samples or suboptimal solutions (Singh Khanuja and Kaur Khanuja 2021).

A primary source of instability is discriminator overfitting. This occurs when the discriminator memorizes specific training examples rather than learning the general features that define the data distribution (Bai et al. 2022; Karras et al. 2020). When the discriminator becomes too powerful too quickly, it classifies synthetic data as fake with near‐perfect confidence, rejecting generator outputs with probability close to 1 (Goodfellow et al. 2014). This results in vanishing gradients, where the loss function flattens out and fails to provide the generator with the meaningful feedback signals needed to update its parameters (Goodfellow et al. 2014; Wolterink et al. 2020). This is a particular risk in livestock genomics, where datasets often have high‐dimensional features but limited sample sizes (*p >> n*). This data structure makes it easy for the discriminator to distinguish real from fake samples based on trivial artifacts rather than biological patterns, further increasing instability. In contrast, if the generator begins to outperform the discriminator too early, the discriminator's feedback becomes random and uninformative, similarly destabilizing the training process (Ferreira et al. 2024).

To mitigate these imbalances, careful hyperparameter selection and tuning are important. Hyperparameters are user‐defined settings that govern how the model is trained rather than parameters learned directly from the data. Examples include the learning rate, batch size, optimizer settings, and the relative number of discriminator and generator updates. In GANs, these settings are especially important because they directly influence the balance between the two competing networks (Mhawi et al. 2024). Goodfellow et al. (2014) described an alternating training strategy in which the discriminator may be updated *k* times for each generator update. The appropriate value of *k* depends on the GAN formulation. For example, the original WGAN implementation used five critic updates (*k* = 5) per generator update to improve optimization of the Wasserstein objective (Arjovsky et al. 2017). The learning rate also affects the magnitude of parameter updates, and relatively small values, such as 0.0002 or lower, have frequently been used to promote stable training (Ferreira et al. 2024; Radford et al. 2016). Pretraining the discriminator on real data for a limited number of epochs has also been proposed to provide a more informative learning signal during the early stages of training (Hiriyannaiah et al. 2020). However, if not carefully tuned, these hyperparameters can allow one network to learn substantially faster than the other, reinforcing the imbalance that contributes to unstable GAN training.

Beyond hyperparameter tuning, the choice of loss function is critical for stability (Yıldız et al. 2024). Several GAN objectives and optimization via loss functions have been developed to mitigate common GAN training issues such as mode collapse, non‐convergence, and vanishing gradients, which can hinder GAN performance and the quality of GAN‐generated data. These variations in GAN objectives result in multiple loss functions, which are discussed and compared in the Section 2.3.

#### 2.2.2 Mode Collapse

Beyond instability, one important challenge in GAN training is mode collapse, in which the generator produces a limited set of repetitive samples rather than capturing the full diversity of the target distribution (Ferreira et al. 2024). In livestock genomics, this is particularly detrimental as it results in synthetic datasets that fail to reflect population‐level variance, including rare alleles, minor allele frequencies, and complex linkage disequilibrium patterns within populations (Saxena and Cao 2020). Consequently, the generated data lose their biological validity, reducing their utility for downstream applications in population and quantitative genetics studies. Mode collapse typically occurs when the generator identifies a specific output pattern that successfully deceives the discriminator. Instead of learning the entire distribution, the generator optimizes for this safe solution, mapping diverse points in the latent space to a single output mode (Srivastava et al. 2017). As the discriminator learns to reject this specific mode, the generator may shift to another single mode, resulting in a cycle where the model never captures the global distribution (Metz et al. 2016; Singh Khanuja and Kaur Khanuja 2021; Wiatrak et al. 2019).

To mitigate this, minibatch discrimination was proposed by Salimans et al. (2016), allowing the discriminator to evaluate a batch of generated samples collectively rather than individually. This enables the discriminator to identify and penalize batches that lack diversity and forces the generator to produce a more diverse output distribution (Salimans et al. 2016). More recently, architectures employing multiple generators trained against a single discriminator have been developed to ensure broader coverage of the data distribution and improved representation of heterogeneous populations (Mohebbi Moghaddam et al. 2023). These strategies are crucial for livestock applications, where preserving the full spectrum of genetic variation is as important as ensuring that the generated data accurately reflects the underlying biological measurements.

#### 2.2.3 Evaluation Metrics for Synthetic Omics Data

Even when training achieves stability and avoids mode collapse, validating the quality of GAN outputs remains a major challenge due to the absence of universal and interpretable metrics (Salimans et al. 2016). Unlike likelihood‐based models, GANs do not provide explicit measures of data probability, limiting the direct interpretability of their outputs (Saxena and Cao 2020). While computer vision researchers rely on established metrics such as the Inception Score (Salimans et al. 2016) and the Fréchet Inception Distance (Heusel et al. 2017) to quantify image realism generated by GANs, these metrics are not directly applicable to structured data like genotype, abundance, or expression matrices. Consequently, the evaluation of synthetic omics data relies on domain‐specific methodologies, often categorized as qualitative and quantitative approaches.

Qualitative approaches typically rely on visual comparisons of real and synthetic data to assess the preservation of biological patterns. For example, principal component analysis is widely used to reveal whether synthetic genotypes capture the population structure and clustering observed in real datasets (van Waaij et al. 2023). Similarly, linkage disequilibrium decay plots, which track the decline of correlation (*r* ^2^) between single nucleotide polymorphisms as a function of physical distance, provide visual confirmation that the synthetic genotype data preserve the dependency structures of the authentic population (Rogers 2013). Additionally, minor allele frequency histograms provide another key qualitative assessment; similar distributions between real and generated datasets suggest that synthetic genotypes capture the natural levels of genetic diversity (Rezaei and Hedayat 2013).

Quantitative approaches, however, employ statistical tests to provide objective measures of similarity. Hypothesis testing frameworks, including permutation tests, *t*‐tests, and the Kolmogorov–Smirnov (KS) test, are used to assess whether the synthetic and real distributions differ significantly (Oprisanu et al. 2022). Furthermore, correlation analyses of linkage disequilibrium statistics or allele frequencies provide metric‐based evidence of alignment; high correlation coefficients between the real and synthetic matrices (i.e., linkage disequilibrium and allele frequencies) indicate an effective reproduction of genomic patterns (Shi et al. 2022; Yelmen et al. 2021). These qualitative and quantitative analyses provide a holistic framework for assessing the quality of GAN‐generated genotype data. Such evaluation metrics are especially important in livestock when generating genotypes, where synthetic genotypes must not only be realistic but also preserve the population genetics patterns and remain statistically reliable for downstream applications.

### 2.3 Loss Function

Loss functions define the optimization objectives that guide GAN training and determine the gradient signals used to update the generator and discriminator (Pan et al. 2020; Souza et al. 2023). In the adversarial framework, the discriminator is optimized to distinguish real observations from GAN‐generated samples, whereas the generator is optimized to produce samples that increasingly resemble the real data and are therefore more difficult for the discriminator to identify (Goodfellow et al. 2014). The choice of loss function is important because it determines not only what constitutes an error during training but also how strongly the networks are penalized for that error. Consequently, different loss formulations can provide substantially different learning signals even when the underlying generator and discriminator architectures remain unchanged. This distinction is particularly relevant during early training, when the generated and real data distributions may differ considerably.

The original GAN formulation uses a binary cross‐entropy objective in which the discriminator learns to classify observations as real or generated (Goodfellow et al. 2014). Under an optimal discriminator, this minimax formulation can be related to minimizing the Jensen‐Shannon divergence between the real and generated data distributions (Sinn and Rawat 2017). The Jensen‐Shannon divergence measures dissimilarity between two probability distributions by comparing each with their average distribution (Lin 1991). According to the authors, minimizing this divergence should bring the generated distribution closer to the real distribution. A practical limitation arises, however, when the two distributions have little overlap (Arjovsky et al. 2017). In this situation, the discriminator can readily separate real from generated samples and become highly confident in its predictions (Yoo et al. 2020). The resulting objective may provide very small gradients to the generator, limiting the information available for improving its outputs and contributing to unstable training (Saxena and Cao 2020). Goodfellow et al. (2014) partly addressed this limitation with the non‐saturating generator objective, which maximizes the log‐probability that generated samples are classified as real rather than directly minimizing the original minimax objective. Although both objectives have the same theoretical solution, the non‐saturating formulation provides stronger gradients when generated samples are easily identified as synthetic (Goodfellow et al. 2014).

This limitation becomes particularly relevant for high‐dimensional data because the real and generated distributions may occupy substantially different regions of the data space during early training (Zuo et al. 2022). Rather than asking the discriminator only to classify observations as real or generated, the Wasserstein GAN (WGAN) changes the optimization problem by estimating how far the generated distribution is from the real distribution (Arjovsky et al. 2017). WGAN uses the Wasserstein distance, which can be interpreted as the minimum cost required to transform one probability distribution into another (Arjovsky et al. 2017; Okano and Imaizumi 2022). In this framework, the discriminator is replaced by a critic that assigns continuous scores to real and generated samples instead of class probabilities (Song and Ermon 2020). The key practical difference is that the Wasserstein distance can provide an informative measure of discrepancy even when the two distributions have little or no overlap (Jin et al. 2020). Thus, generated samples that remain far from the real distribution can still receive meaningful gradient information, allowing the generator to continue improving instead of encountering the gradient saturation associated with the original GAN objective (Saqlain et al. 2021; Wolterink et al. 2020). This property generally produces smoother optimization and makes the loss value more informative about training progress (Wali et al. 2022).

The benefit of the Wasserstein objective depends on constraining the critic to satisfy the Lipschitz condition, which limits how rapidly its output can change in response to changes in the input (Arjovsky et al. 2017; Zhou et al. 2019). The original WGAN enforced this condition by clipping the critic weights to a predefined range. Although computationally straightforward, weight clipping can restrict the capacity of the critic and lead to undesirable optimization behavior, including poor gradient propagation (Gulrajani et al. 2017). WGAN with gradient penalty (WGAN‐GP) addresses this limitation by replacing weight clipping with an additional penalty applied when the norm of the critic gradient deviates from the desired value (Park et al. 2021). In practice, this penalty is evaluated for samples interpolated between real and generated observations, encouraging the critic to change smoothly across the region connecting the two distributions (Gulrajani et al. 2017). Thus, WGAN‐GP does not fundamentally change what WGAN optimizes, but it changes how it enforces the constraint required for Wasserstein optimization. This generally produces more stable gradients and avoids some of the capacity restrictions introduced by weight clipping, although calculating the gradient penalty increases computational cost (Yıldız et al. 2024).

Other loss functions address similar training limitations through different mechanisms. The least‐squares GAN (LSGAN), introduced by Mao et al. (2017), replaces the binary cross‐entropy objective with a least‐squares loss. Under the original classification‐based objective, generated samples that fall on the synthetic side of the discriminator boundary with high confidence may provide relatively weak gradients (Mao et al. 2017). LSGAN instead penalizes samples according to their distance from the desired discriminator output (Yıldız et al. 2024). Consequently, generated samples located far from the decision boundary continue to incur a substantial penalty, providing the generator with an informative gradient that encourages movement toward regions occupied by real observations (Mao et al. 2017). This modification has been associated with more stable optimization and is related to minimizing the Pearson $\chi^{2}$ divergence (Mao et al. 2017; Yıldız et al. 2024). Loss‐Sensitive GAN (LS‐GAN) uses a different strategy by imposing a margin between the losses assigned to real and generated samples. Real samples are encouraged to receive lower losses than generated samples by a predefined margin, while the generator attempts to reduce the loss of its generated observations (Qi 2018). Instead of relying exclusively on binary classification, this formulation uses relative loss differences to provide the learning signal and was proposed to improve generalization of the generated distribution (Qi 2018).

These loss functions, therefore, differ primarily in how they quantify disagreement between real and generated data and how that disagreement is translated into gradients for optimization. The original adversarial loss provides a probabilistic real vs. generated classification objective but may suffer from weak generator gradients when the discriminator becomes highly confident. LSGAN modifies the shape of this penalty such that poorly generated samples continue to receive informative gradients. WGAN moves away from binary classification and instead optimizes a continuous measure of distributional discrepancy, whereas WGAN‐GP improves the practical enforcement of the constraint required for that objective. No single loss function is universally optimal because performance depends on the architecture, training configuration, data dimensionality, sample size, and characteristics of the target distribution (Dong and Yang 2019; Lucic et al. 2018; Pan et al. 2020). For omics applications, this choice is particularly important because the objective must support stable training while allowing the generator to reproduce both common and less frequent biological patterns. In genomic data, for example, inadequate optimization or loss of distributional diversity could result in synthetic datasets that reproduce common genotypes while poorly representing rare alleles, haplotypes, or population substructure.

### 2.4 Final Considerations

GANs are emerging as a useful framework in biological research, but the challenges discussed above highlight the complexity of applying them in practice. Issues of instability, mode collapse, and the lack of universal evaluation metrics remain active barriers to widespread adoption. These limitations are magnified in livestock genomics, where synthetic data are expected to reproduce key genetic and molecular patterns observed in real datasets, including allele frequency spectra, linkage disequilibrium, diversity across individuals or groups, and expression and co‐expression structures. Consequently, the ideal model must be stable enough to converge, diverse enough to represent rare variants, and rigorously validated to ensure these patterns are accurately reproduced. Overcoming these barriers will require continued methodological innovation and careful adaptation of existing GAN frameworks to the unique properties of livestock omics data. Addressing these constraints is critical to unlocking the full potential of GANs for generating realistic, interpretable, and actionable synthetic data in livestock and beyond.

## 3 Software Frameworks and Computational Considerations

GANs can be implemented using general‐purpose deep learning frameworks, with PyTorch (Paszke et al. 2019) and TensorFlow (Abadi et al. 2016) among the most widely used platforms. These frameworks provide the neural‐network layers, automatic differentiation, optimization algorithms, and hardware acceleration required to construct and train the generator and discriminator. PyTorch provides a flexible framework for defining custom adversarial training loops, whereas TensorFlow and Keras offer both lower‐level model customization and higher‐level interfaces for implementing GAN architectures. Implementations of commonly used GAN variants, including conventional GANs, conditional GANs, and WGAN‐GP, are available within these ecosystems and can be adapted to genomic, transcriptomic, and other omics data structures. In practice, implementation requires specifying the generator and discriminator architectures, loss functions, optimization algorithms, and training hyperparameters, followed by domain‐specific evaluation of the generated data.

The computational resources required for GAN training depend strongly on the dimensionality of the input data, network architecture, batch size, number of training iterations, and extent of hyperparameter tuning. Small tabular omics datasets and relatively simple fully connected architectures may be trained using modest computational resources, whereas high‐dimensional genotype matrices, sequencing data, and more complex convolutional architectures can substantially increase memory and processing requirements. Graphics processing units (GPUs) are commonly used to accelerate the matrix operations involved in deep neural‐network training, although GPU acceleration may provide limited benefit for very small models or datasets. For larger applications, GPU memory can become an important constraint because model parameters, intermediate activations, gradients, and batches of high‐dimensional omics observations must be retained during training. Dimensionality‐reduction approaches, smaller batch sizes, or distributed training across multiple GPUs can be considered when the full dataset or model cannot be efficiently accommodated on a single device.

Computational efficiency can also be improved through mixed‐precision training, in which selected operations are performed using lower‐precision numerical formats while operations requiring greater numerical stability retain higher precision. This approach can reduce memory requirements and accelerate training on compatible GPU hardware. However, because GAN training is sensitive to numerical stability and hyperparameter configuration, computational efficiency should not be prioritized at the expense of convergence or quality of the generated data. For reproducibility, studies applying GANs to omics data should report the software framework and version, hardware configuration, model architecture, batch size, optimizer and learning rates, number of training iterations or epochs, random seeds, and relevant computational strategies such as mixed‐precision or multi‐GPU training.

## 4 Generative Adversarial Networks Applications for Omics Data

Generative Adversarial Networks (GANs) have been applied across diverse domains, ranging from generating photorealistic human faces (Fang et al. 2022) and assisting in COVID‐19 diagnostics (Gulakala et al. 2022) to detecting mechanical defects in industrial machinery (Liao et al. 2024) and monitoring structural integrity in civil engineering projects (Luleci et al. 2022). Beyond these engineering and diagnostic applications, GANs have been explored as an alternative for generating synthetic omics data that preserve the statistical and structural properties of real datasets (Lee 2023). Compared with other generative models, GANs have demonstrated superior performance in producing realistic synthetic samples without relying on explicit likelihood estimation (Balaji et al. 2019; Grover et al. 2018). These synthetic datasets are valuable for augmenting training data, mitigating privacy constraints, and addressing class imbalance in biological datasets (Yelmen et al. 2021).

The architecture of GAN is well‐suited to omics data modeling. For example, genotype matrices, where rows represent samples and columns represent single nucleotide polymorphisms (SNP), can be processed similarly to high‐dimensional image data. Convolutional layers, widely used in computer vision, have been successfully adapted to extract biologically relevant patterns from these matrices, generating synthetic outputs that observed genomic structure and variation (Riley et al. 2024). This section reviews recent applications of GANs across multiple omics domains, including transcriptomics, single‐cell RNA sequencing, and whole‐genome sequence data. The following subsections focus on how these models have been applied to augment genomics studies, enhance privacy data sharing, mitigate sampling biases, and support population genomics inference. While GANs have also shown promising results for multi‐omics data integration (Afroz et al. 2024; Ahmed et al. 2021; Bahrami et al. 2021), this review focuses specifically on single‐omics applications. Such an overview of current GAN applications provides a roadmap for their future adoption in livestock omics.

### 4.1 Population Genomic Studies

Simulation‐based approaches are important in evolutionary biology and population genetics, providing a framework for testing hypotheses under controlled and replicable conditions (Trost et al. 2024). Traditional simulations, however, require explicit assumptions regarding biological parameters, such as mutation rates, recombination frequencies, migration models, and selection coefficients. These parameters are often difficult to estimate accurately or may fail to capture the full complexity of natural populations (Booker et al. 2023). As a result, conventional simulations risk introducing biases and limiting the generalizability of their outcomes.

To address this challenge, Wang et al. (2021) developed pg‐GAN, a GAN‐based framework designed to synthesize genomic data under a parameterized demographic model. Unlike conventional simulators that require fixed inputs, pg‐GAN learns evolutionary parameters directly from empirical data. In this architecture, the generator acts as a parameter proposer, outputting demographic values that are fed into a coalescent simulator to produce synthetic genotype matrices. The discriminator then evaluates whether these simulated datasets resemble real genomic samples. Through adversarial training, the generator adjusts its proposed parameters to maximize the similarity between synthetic and real data, effectively performing likelihood‐free inference. This method enables the generation of realistic genomic datasets with minimal manual parameter specification and is particularly promising for non‐model organisms, where demographic parameters are often uncertain or unavailable. Building on this framework, Riley et al. (2024) applied pg‐GAN to simulate genomic regions evolving under selective pressures. In their implementation, the discriminator was trained to distinguish neutral regions from those under natural selection, allowing the generation of synthetic genomic sequences that exhibit the specific patterns of signatures associated with selective sweeps. This innovation provides a powerful tool to benchmark selection detection methods, accelerating our understanding of adaptive evolution.

Another generative framework called MutaGAN, proposed by Berman et al. (2020), combines a GAN framework with a sequence‐to‐sequence recurrent neural network to simulate protein sequence evolution and predict potential mutations. When applied to influenza virus proteins, MutaGAN generated variants with a median Levenshtein distance of 2.00 from real sequences, indicating strong biological realism. Results show that 72.8% of the generated proteins contained at least one mutation already observed in the global influenza virus population, highlighting the model's ability to simulate realistic mutational dynamics. This has significant implications for viral evolution studies, supporting vaccine development and informing pandemic preparedness strategies. These examples demonstrate the potential of GAN‐based approaches to transform the field of population genomics. Moving from assumption‐based models to data‐driven learning allows GANs to learn directly from empirical data, capturing complex evolutionary patterns and generating biologically meaningful synthetic datasets. These advances open new opportunities for investigating demographic history, adaptation, and genomic variation across a wide range of livestock species.

### 4.2 Artificial Genomes

Generating synthetic genomes using GANs is a promising approach to addressing challenges in genomics research, including population imbalance, limited sample sizes, and data privacy concerns. One of the earliest studies to generate synthetic genomes was conducted by Yelmen et al. (2021), who combined a GAN model with Restricted Boltzmann Machines (RBM) to generate artificial human genomes using data from the 1000 Genomes Project. Their model successfully captured key features of the human genome, including linkage disequilibrium patterns and haplotype structure, while maintaining high fidelity to real genomic data. The synthetic genomes achieved correlations of 0.95–0.97 with the real datasets across two training cohorts and preserved population‐specific genomic patterns, particularly from underrepresented groups of ancestries. The generated samples were also unique and did not replicate any individual from the training dataset, offering an alternative framework for conducting downstream analysis by increasing reference panels with synthetic genomic data without compromising individual privacy (Yelmen et al. 2021). A second study from the same group, Yelmen et al. (2023) further improved upon this approach by comparing a convolutional Wasserstein GAN (WGAN), variational autoencoders (VAE), and conditional RBM for large‐scale genomic data generation. These models demonstrated improved preservation of allele frequency distributions and population structure, increasing the usefulness of synthetic genomes in population genomics and disease studies.

Chen et al. (2020) demonstrated that GAN‐based approaches generated realistic genotypes while preserving biological structure in highly polymorphic regions, such as the human leukocyte antigen (HLA) locus. The HLA region is extremely diverse among individuals and populations due to its role in the adaptive immune response (Noble and Valdes 2011). In their study, Chen et al. (2020) used 7160 SNPs from 2504 individuals representing five subpopulations: Europeans, East Asians, Africans, Americans, and South Asians from the 1000 Genome Project. Their model, called PG‐cGAN, employed a conditional Wasserstein GAN architecture stacked with 1D convolutional layers. The conditional framework enables the generator and critic to generate population‐specific genomic features of each subpopulation, despite class imbalance. The results showed strong alignment between the generated and real genotypes, particularly in capturing biological features like population structure, allele frequencies, and linkage disequilibrium. Additionally, the model achieved a low mean squared error (MSE) below 0.01 for the linkage disequilibrium pattern between the real and generated genotypes.

Das and Shi (2022) introduced Offspring‐GAN, a model based on PG‐cGAN that adds conditional constraints to address the limitations of existing datasets, which often underrepresent specific ethnic groups. Offspring‐GAN was designed to generate synthetic genomic data for underrepresented groups in biased datasets while minimizing mode collapse. The model incorporates subsampling strategies that guide the generator toward synthesizing minority classes and implements discriminator‐side constraints to maintain genomic diversity. The Privacy‐Preserving Offspring GAN (PPO‐GAN), a variation of the Offspring‐GAN, was also proposed by Das and Shi (2022) to generate synthetic data exclusively for underrepresented classes. Both models outperformed PG‐cGAN in preserving key genomic structures such as population structure and minor allele frequency. The similarity between Gaussian component means and weights observed for Offspring‐GAN was 0.988, whereas for PPO‐GAN it was 0.980, compared to 0.741 from PG‐cGAN, suggesting that the generated samples improved preservation of population genetic structure and retained sufficient biological validity to be used in downstream tasks without compromising data privacy (Das and Shi 2022). Ahronoviz and Gronau (2024) proposed another conditional GAN architecture, called AC‐GAN, designed to model genomic substructure within large‐scale datasets. In this design, the generator receives a conditional class label, while the discriminator predicts both the class label and the origin (real or synthetic) of each sample. This forces the model to learn a more detailed intra‐population structure. Furthermore, samples generated by AC‐GAN improved mean classification accuracy across multiple predictive models, including a support vector classifier, K‐nearest neighbors models with K = 5, and a six‐layer neural network with a limited number of training samples. These results suggest that GAN‐generated genomic data can improve the performance of downstream analysis (Ahronoviz and Gronau 2024).

GAN‐based data augmentation has shown potential to increase the representation of underrepresented classes in small genomics datasets and to support semi‐supervised prediction. Davi and Braga‐Neto (2021) introduced gGAN, a semi‐supervised model designed to generate synthetic genetic profiles and predict genetic predisposition to severe dengue using genomic data from a Brazilian population. In this model, the discriminator had a dual function: first, to distinguish real from generated genomic profiles, and second, to classify genomes by their susceptibility to dengue. When evaluated on held‐out unlabeled profiles that were known to be real, the best‐performing model correctly recognized 94.3% as real, indicating that these genetic profiles were sufficiently consistent with the genomic distributions represented during training. A real profile classified as synthetic was interpreted by the authors as potentially originating from a population insufficiently represented by the training data and, consequently, as less suitable for disease‐severity prediction. Among profiles considered compatible with the training population, the model achieved 93.75% accuracy in predicting dengue severity. Thus, in this application, the discriminator's real vs. generated output served not only the adversarial training process but also as an additional measure of whether a new genomic profile was appropriate for prediction. This study illustrates how GAN architectures can be extended beyond synthetic data generation to combine semi‐supervised learning with genomic prediction and an assessment of population compatibility. Murad et al. (2023) also explored the use of GAN models to augment genomic data, with a focus on pathogen genomics. Their GAN model was designed to improve classification performance in scenarios involving imbalanced datasets, such as those rare pathogen strains. Although originally tailored for microbial sequence analysis, the architecture is generalizable to other biological domains, including livestock and human genomics.

GANs have also been applied to variant calling, a core task in genomic sequence data processing that involves identifying single nucleotide variants or indels. Yang, Gu, et al. (2022) developed LDV‐caller, a GAN‐based model for variant calling using low‐depth sequencing data. Low sequencing coverage often reduces variant‐calling accuracy; however, LDV‐caller was trained to reconstruct accurate genotypes from noisy, low‐depth inputs. When validated on two benchmark datasets, Genome in a Bottle and SARS‐CoV‐2, the model achieved F1‐scores of 92.77% and 94.25%, respectively. For comparison, high‐depth sequencing on the Genome in a Bottle dataset achieved an F1‐score of 94.49%, indicating that GAN models may offer a cost‐effective alternative for accurate variant calling from low‐coverage sequencing data with minimal performance loss. These studies demonstrate that GANs offer a flexible and powerful framework for generating artificial genomic data. Whether through the synthesis of entire genomes, the augmentation of underrepresented classes, or the enhancement of classification and variant calling performance, GANs are well‐suited to address key challenges in genomic data analysis, including data privacy preservation, sample imbalance, and the generation of synthetic high‐throughput data.

### 4.3 Transcriptomic Studies

Transcriptomic data from RNA sequencing (RNA‐seq) are crucial for understanding gene expression and its role in phenotype variation. However, high sequencing costs and limited sample availability often constrain the application of traditional and deep learning approaches in this domain, reducing the statistical power of conventional analyses and limiting the large training sets typically required for deep learning models (Lee 2023). To overcome this, researchers have increasingly employed GANs to augment transcriptomic datasets to improve downstream predictive analyses. Lacan et al. (2023) trained multiple GAN models on data from The Cancer Genome Atlas to enhance cancer phenotype prediction. The GAN augmentation technique significantly improved prediction accuracy for cancer detection and for predicting the tissue of origin of mRNA samples based on transcriptomic data. Using 50 real RNA‐seq samples, the baseline model achieved 94% accuracy for cancer detection and 70% accuracy in predicting tissue origin. With GAN‐augmented data, cancer prediction accuracy increased to 98% and tissue of origin prediction to 94%, respectively, highlighting the potential of GANs to enhance classification performance in scenarios with limited sample size.

GANs have also been employed to improve the statistical power of transcriptomic analyses, particularly in detecting differentially expressed genes. Waters et al. (2024) developed GAiN, a GAN‐based model designed to increase the number of RNA‐seq samples in datasets with fewer than 20 samples per phenotype, enabling robust differential expression and pathway analysis. In cancer experiments to differentiate between lung adenocarcinoma (LUAD) and lung squamous cell carcinoma (LUSC), baseline analysis recovered 602 of 1000 true differentially expressed genes (DEGs), while GAiN‐based analysis recovered 800 of 1000 true DEGs. Moreover, GAiN successfully reproduced five of the six enriched pathways that were biologically distinct between the two cancer phenotypes. These results suggest that GAiN may implicitly learn and replicate biologically meaningful relationships embedded in the training data. Complementing this work, Wang et al. (2024) used a baseline GAN with a minimax normalization approach to improve the representation of transcriptomic data. The generated synthetic transcriptomes showed low divergence from real data, achieving maximum mean discrepancy (MMD) values of 0.030 and 0.033 for the training and testing datasets, respectively.

Viñas et al. (2022) implemented a Wasserstein GAN with gradient penalty (WGAN‐GP) to generate synthetic transcriptomic data for *Escherichia coli* . Their model outperformed traditional simulators such as SynTReN and GeneNetWeaver in preserving biological properties relevant to gene regulatory networks. Furthermore, WGAN‐GP was applied to human transcriptomic data from The Cancer Genome Atlas and the Genotype‐Tissue Expression project, successfully conserving biological clustering structures and expression dynamics across diverse tissue and cancer types. These findings suggest that the model can capture complex gene expression patterns and regulatory interactions from a systems biology perspective.

In earlier work, Wang et al. (2018) developed a conditional GAN architecture designed to infer genome‐wide gene expression profiles from a small set of landmark genes. Landmark genes are a subset of genes whose expression patterns account for most of the transcriptional variability and can accurately predict the expression of thousands of related genes (Clayman et al. 2021). Their model incorporated adversarial training with an L1‐norm loss function to stabilize learning and was validated using publicly available transcriptomic datasets from Gene Expression Omnibus and Genotype‐Tissue Expression databases. The inclusion of GAN‐generated synthetic data enhanced the reconstruction of gene profiles from partial measurements, demonstrating the application of GANs models to extend gene regulatory networks. Similarly, Dizaji et al. (2018) proposed a semi‐supervised GAN model trained using expression profiles limited to a set of landmark genes. This approach, validated on Gene Expression Omnibus and Genotype‐Tissue Expression microarray datasets, improved genome‐wide expression inference with only a small number of labeled examples, confirming its effectiveness in scenarios with limited sample size.

Targonski et al. (2019) developed a GAN approach that accurately simulates gene expression perturbations across different cell states. The Transcriptome State Perturbations Generator (TSPG) was trained on the Genotype‐Tissue Expression and The Cancer Genome Atlas datasets to simulate patient profiles with different tumors and cell‐state progression. The model demonstrated remarkable performance, as 99% of generated samples were classified as real. Moreover, the model was not only capable of generating plausible perturbations across multiple tissues but also of identifying gene‐level changes across tissues, with 85 genes consistently upregulated and 642 genes downregulated. In addition, pathways enriched analysis revealed between 933 and 1522 enriched pathways, demonstrating the potential of GANs to capture complex transcriptomic perturbations and regulatory dynamics in biology.

Zrimec et al. (2022) developed ExpressionGAN, a GAN‐based pipeline for synthetic engineering of DNA regulatory regions with enhanced expression control. Their approach first trained a GAN to generate synthetic regulatory regions from natural sequences and then evaluated the generated regions using a predictive model. The results showed that ExpressionGAN‐generated sequences could alter gene expression levels by approximately 57% compared to natural regulatory regions, offering new tools for regulatory genomics and synthetic biology. In the context of cancer transcriptomics, Farou et al. (2020) introduced the Gene Expression Generator (GEG), a WGAN‐based framework for simulating cancer transcriptomic profiles. The model demonstrated increased classification accuracy for cancer detection, particularly for breast cancer, boosting accuracy by more than 15% when classifiers were trained on both real and synthetic data, supporting the role of GAN in enhancing sample diversity and improving predictive modeling. Jahanyar et al. (2023) reported that GAN models are suitable for analyzing microarray transcriptomic data. Their work focused on increasing the number of samples for microarray transcriptomic data on schizophrenia using a model called MS‐ACGAN, which outperformed other GAN variants, such as ACGAN, in terms of model reliability, data quality, and diversity.

MicroRNA (miRNA) is a class of non‐coding RNA molecules with an approximate 20‐nucleotide length that regulate gene expression at the post‐transcriptional level, typically by binding to complementary sequences on target mRNAs and promoting their degradation or inhibiting translation (Ojo and Kreuzer‐Redmer 2023). Due to their central role in apoptosis and cell cycle regulation, miRNAs are also key players in cancer development and progression (Sumaira et al. 2024). Tamilmani et al. (2022) developed a GAN‐based approach integrated with optimization techniques to enrich miRNA datasets for cancer biomarker discovery. By balancing class distribution through synthetic data generation, their model achieved 99.26% accuracy, with sensitivity and specificity of 95.23% and 92.56%, respectively. These results significantly outperformed standard machine learning approaches, demonstrating the added value of GAN models in miRNA‐based classification and biomarker identification. In summary, these studies illustrate the broad applicability of GAN models in transcriptomics, from RNA‐seq augmentation and differential expression analysis to regulatory sequence engineering and miRNA biomarker discovery. GAN‐based models enable researchers to overcome sample‐size limitations, preserve biological signals, and enhance downstream analytical tasks, serving as a valuable complement to existing computational methods in functional genomics.

### 4.4 Single‐Cell RNA Sequences

Single‐cell RNA sequencing (scRNA‐seq) has revolutionized transcriptomic analysis by enabling the profiling of gene expression at single‐cell resolution (Baysoy et al. 2023). This allows for the study of cellular heterogeneity, lineage trajectories, and gene regulatory mechanisms across different tissues and physiological conditions (Wu et al. 2024). However, scRNA‐seq data present analytical challenges in identifying rare cell populations because of their low abundance, which might limit sensitivity, particularly in cancer diagnostics and disease progression (Jindal et al. 2018). This limitation can hinder accurate modeling of gene expression dynamics and regulatory interactions (Jin et al. 2024).

To address these limitations, Ghahramani et al. (2018) demonstrated the applicability of GANs for integrating scRNA‐seq datasets derived from different cell types and laboratory protocols. Their model, which used a conditional GAN to model cell type and perturbation status, was capable of generating both the overall population structure and distinct cellular subpopulations, despite technical variability across data sources. A key innovation involved latent space interpolation, where the generator learned to map gene expression profiles in the latent space, such that small changes across the latent space would reflect changes in cellular states. This enabled the researchers to model cell‐state transitions and perturbations. Consequently, the framework simulated cellular perturbations and reconstructed transcriptomic responses with high temporal resolution. Furthermore, analysis of the generator's internal weights revealed co‐expression patterns and potential gene regulatory relationships, highlighting the utility of GANs for uncovering biological mechanisms in the latent representation of complex single‐cell data.

Building on the integration of generative frameworks, Yu and Welch (2021) developed MichiGAN, a hybrid model that combines GAN and variational autoencoder (VAE) strategies to generate scRNA‐seq data and simulate perturbations under different biological scenarios. MichiGAN uses a Beta‐total correlation variational autoencoder (β‐TCVAE) to separate sources of variation in the latent space, and a conditional WGAN‐GP to generate synthetic scRNA‐seq data. MichiGAN was able to simulate perturbations because, unlike traditional VAEs where all the features and biological signals are often mixed within the same variables in the latent space, β‐TCVAE enforces separation of the biological signals by penalizing the total correlation among latent dimensions, which ensures that features such as cell identity and drug treatment are in different dimensions. These independent dimensions serve as conditional inputs for the WGAN‐GP generator. By separating biological signals into independent axes, researchers performed “virtual experiments” by modifying the latent coordinates associated with a specific perturbation (e.g., drug treatment) while holding the other variables constant (cell type). WGAN‐GP then learns the manipulated latent features associated with scRNA profiles and generates scRNA‐seq profiles corresponding to different cellular states. This demonstrated the potential of generative models to predict transcriptional responses under unobserved conditions, which can accelerate in silico drug screening and cellular reprogramming studies.

Tsourtis et al. (2022) proposed a semi‐interpretable GAN architecture to better capture scRNA‐seq distributions, while addressing issues such as sample imbalance and zero inflation due to technical dropout and low transcript detection without requiring extensive preprocessing. In addition to the regular black‐box fully connected generator, they introduced two probabilistic generators to improve the interpretability of the generative process: a Gaussian Mixture Model (GMM) generator and a Zero Inflated Mixture Model (ZIMM) generator. GMM generator, unlike traditional generators, modeled the data distribution as a weighted combination of multiple Gaussian components. The Gaussian parameters were estimated iteratively during training by optimizing the model to capture the statistical structure of the real scRNA‐seq distribution. During training, the GMM estimated the mean, covariance, and relative contribution of each Gaussian component to approximate the Gaussian components of the real data. The mean represented the average gene expression profile of a subpopulation, the covariances described the relationship and variability among genes, and the mixture weight reflected the relative abundance of each component within the dataset. Because the synthetic scRNA dataset parameters were based on the real population distribution, researchers could interpret different regions of the transcriptomic distribution as potential cellular subpopulations or transcriptional states, improving biological interpretation of the generative process.

The ZIMM generator was designed for scRNA‐seq datasets, which often contain zero inflation because of sparse gene expression and low transcript capture efficiency. In a standard generator, gene expression is modeled as a continuous process, which poses a challenge to capture the zero‐inflated values characteristic of scRNA‐seq data. To tackle this, the ZIMM generator implemented a two‐stage probabilistic framework to separate gene expression presence from gene expression magnitude. First, a Bernoulli distribution was used to estimate whether a gene was expressed, capturing the zero‐inflated structure commonly observed in single‐cell datasets. Second, conditional on expression, a Gaussian mixture model was used to estimate the magnitude and variability of expressed genes. These Gaussian components modeled heterogeneous gene expression patterns across cellular populations and transcriptional states. While modeling zero‐inflated data and gene expression values independently, ZIMM faithfully captured the sparse and zero‐inflated structure of scRNA‐seq, preserving biologically meaningful gene expression patterns and magnitudes. This approach improved interpretability because the Bernoulli components can be interpreted as gene activation status, while the Gaussian components estimated the magnitude and variability across cells. Based on maximum mean discrepancy (MMD) values, histograms, and PCA comparisons, the fully connected neural network demonstrated robust performance (lowest MMD, most realistic generated data) across various datasets. The ZIMM generator was more sensitive to hyperparameter tuning but excelled in capturing rare cell types when optimally configured. Their work highlights the importance of balancing model interpretability with generative accuracy in single‐cell analyses.

More recently, Zinati et al. (2024) developed GRouNdGAN, a GAN‐based framework that integrates prior knowledge from gene regulatory networks to simulate realistic scRNA‐seq data. This model enables the user to define input features that shape the internal representation of cell states, explicitly accounting for transcription factor control. Unlike models that assume batch effects or homogeneous cell populations, GRouNdGAN permits user‐defined modeling of heterogeneous or admixed samples, without imposing strong parametric assumptions. While this flexibility allows researchers to simulate complex differentiation or perturbation scenarios, it requires a foundational understanding of the biological system being modeled to configure simulation parameters effectively. These studies demonstrate the growing potential of GAN‐based approaches to address fundamental challenges in scRNA‐seq data analysis. From integrating datasets across batches and laboratories to modeling cell differentiation and simulating transcriptional responses, GAN provides a data‐driven and biologically interpretable framework for single‐cell genomics. As scRNA‐seq technologies continue to advance, GANs are expected to play an increasingly important role in enhancing the resolution and reliability of single‐cell data analyses across diverse biological systems, including livestock genomics.

### 4.5 Proteomics and Protein Design

In proteomics and synthetic biology, GANs have been explored as tools for designing novel proteins with specific biochemical properties (Kucera et al. 2022). These approaches aim to generate biologically functional amino acid sequences, potentially reducing the time and cost associated with traditional protein engineering methods. For instance, Gupta and Zou (2019) introduced the feedback GAN (FBGAN), a framework for generating synthetic DNA sequences that encode proteins with desired characteristics such as antimicrobial activity. FBGAN integrates an external evaluator into the GAN architecture to guide the generator toward producing biologically relevant sequences. The evaluator is also a neural network trained on cDNA sequences encoding known non‐antimicrobial and antimicrobial peptides (AMP), learning physicochemical properties associated with antimicrobial activity such as amino acid composition, charge, hydrophobicity, helicity, and sequence motifs. Initially, the generator produces sequences without bias toward any specific functional property. These sequences are then assessed by the evaluator, who calculates the likelihood that each sequence possesses the target characteristics like antimicrobial activity. Sequences exceeding a predefined probability threshold are selected to optimize the generator's performance. This feedback loop refines the generation process by prioritizing sequences that perform well according to the evaluator. The evaluator achieved an accuracy of 0.842, an area under the receiver operating characteristic curve of 0.908, a precision of 0.826, a recall value of 0.8608, and the area under the precision‐recall curve of 0.88, demonstrating high predictive performance for identifying AMP‐like sequences. The authors applied this framework to design short antimicrobial peptides (fewer than 50 amino acids), demonstrating that feedback GAN could successfully generate protein candidate sequences with functional potential. Furthermore, FBGAN‐generated sequences shared physicochemical properties such as charge, amino acid composition, hydrophobicity, helicity, and aromaticity with the real data, suggesting that FBGAN successfully learned biologically meaningful AMP‐like sequence patterns for AMP generation.

In a related effort, Repecka et al. (2021) developed ProteinGAN, a GAN‐based model trained to generate novel functional proteins using naturally occurring protein sequences as a template dataset. ProteinGAN leverages the sequential patterns and evolutionary signals embedded in protein sequences to synthesize new proteins that resemble naturally occurring ones. The model was tested using malate dehydrogenase as a reference enzyme. Notably, approximately 24% of the ProteinGAN‐generated sequences demonstrated catalytic activity in vitro, indicating that the model could generate proteins with real functional utility. This work represents a significant step toward the use of deep generative models for protein design in synthetic biology, with implications for enzyme engineering, therapeutic development, and industrial biotechnology.

More recently, a generative AI framework called AlphaFold achieved remarkable results when applied to protein structure prediction. For instance, AlphaFold 3 (Abramson et al. 2024) is a diffusion model trained not only to generate proteins but also to predict three‐dimensional protein structure and protein–protein, protein‐DNA, protein‐RNA, and antibody–antigen interactions with higher accuracy than existing state‐of‐the‐art models, such as RoseTTAFold All‐Atom (Abramson et al. 2024). Diffusion models are more stable than GANs and employ a VAE‐like approach (Yang, Srivastava, and Mandt 2022). Diffusion models work by adding noise to the training data until the signal is almost completely obscured and almost indistinguishable from random noise (Ho et al. 2020). The model then reconstructs the original data by iteratively removing noise and restoring meaningful patterns to approximate the real data distribution (Yang, Srivastava, and Mandt 2022). In other words, diffusion models reverse the noise process and generate new data by learning the data distribution during the denoising step, preserving features fundamental to maintaining data structure (Ho et al. 2020). In this sense, AlphaFold 3 operates directly on atomic coordinates and, during the denoising phase, iteratively refines them to generate realistic molecular conformations, resulting in more accurate predictions of atomic interactions, molecular geometries, and biomolecular complexes compared to the previous AlphaFold 2 model (Abramson et al. 2024). These studies highlight how GANs and diffusion models can advance proteomics and protein engineering by enabling the generation of functionally relevant protein sequences. As generative architectures continue to evolve, they may become essential tools for designing the next generation of proteins tailored to specific biomedical applications and for complementing structure‐prediction frameworks such as AlphaFold 3 (Abramson et al. 2024).

## 5 Future Directions of Generative Adversarial Networks in Livestock

GANs might provide new opportunities in livestock genomics research, enabling innovative strategies for data‐driven decisions in breeding, health, and production systems. Their value is unlikely to lie simply in generating additional synthetic observations, but in learning complex empirical distributions that can support specific applications in which livestock data are limited, imbalanced, difficult to share, or expensive to generate. For example, these models could be used to generate synthetic representations of complex omics datasets, augment underrepresented data, support methodological development and benchmarking, and simulate cellular responses to perturbations in silico. GANs can generate synthetic data across various biological scenarios, address data imbalance, and provide controlled datasets for evaluating analytical and predictive approaches. This section explores several emerging applications of GANs in livestock genomics, including the identification of selection signatures, the generation of in silico omics data, the correction of class imbalance in multi‐omics datasets, and the enhancement of model output interpretability. Overall, these developments highlight the potential of GANs as complementary tools for livestock omics research, particularly when their outputs are evaluated according to the biological properties required for the intended application.

### 5.1 Detecting Selection Signatures

Selection signature studies aim to identify genetic variants or loci that increase in frequency because they are associated with economically important traits or adaptation in livestock (Saravanan et al. 2020). Such regions often harbor mutations that drive genetic progress through artificial or natural selection (Demir et al. 2023). However, classical statistical approaches to detect selection signatures often rely on restrictive assumptions, such as constant population size, no admixture, or the absence of recent fixation events (Kumar et al. 2022). Additionally, traditional methods may fail to distinguish between true selection and genomic patterns produced by other evolutionary processes such as balancing selection (Kumar et al. 2022; Soni and Jensen 2024). GANs offer a complementary data‐driven framework for learning complex genomic patterns associated with neutral and non‐neutral evolution. Wang et al. (2021) proposed a GAN architecture in which the generator estimates parameters to simulate genomic sequences across various evolutionary scenarios, making it adaptable to multiple species without requiring prior demographic knowledge. Building on this concept, Riley et al. (2024) trained pg‐GAN to distinguish between genomic regions that evolve neutrally and those under selection. First, pg‐GAN was trained using real genomic data and regions simulated under a neutral demographic model. Then, the trained discriminators obtained from pg‐GAN were fine‐tuned using simulated neutral and selected regions. The fine‐tuned discriminators were then applied across all the real genomic data to identify candidate regions under selection. According to the authors, this framework can be used to identify distinct selected regions across different populations. In livestock, a similar workflow could begin with phased genomic data from a breed or population of interest. A neutral demographic model representing relevant historical processes, such as changes in effective population size, bottlenecks, or admixture, could first be inferred. Genomic regions representing specific selection scenarios could then be simulated and used to fine‐tune the discriminator. The trained discriminator could subsequently be applied across genomic windows from real animals to identify regions that deviate from neutral expectations. Candidate regions could then be examined for genes or QTL associated with economically important traits such as production, efficiency, disease resistance, or fertility. The identified regions should also be compared with established selection‐signature statistics and functional annotations to determine whether the GAN‐based model is detecting biologically plausible signals. In this context, GANs would complement rather than replace established population‐genetic approaches by providing an additional framework for integrating complex genomic patterns associated with selection.

### 5.2 Synthesizing De Novo Omics Data for In Silico Studies

GANs have the theoretical capability to generate realistic synthetic data across diverse biological domains when trained on appropriate and representative datasets. Yelmen et al. (2021) demonstrated that GAN models can simulate artificial genomes that preserve key population‐level genomic features (e.g., linkage disequilibrium and allele frequencies), while generating samples that are distinct from the individuals represented in the training data. These synthetic genomes offer opportunities for methodological and exploratory in silico studies in which realistic genomic distributions are required. Bordukova et al. (2024) emphasized using GANs to generate synthetic biological data under specific conditions for personalized medicine (e.g., disease or treatment scenarios), drawing parallels to the emerging concept of Digital Twins in human medicine. Digital Twins are virtual representations of an individual's biological data, used to simulate treatment responses, optimize medical interventions, and predict disease progression in a personalized manner (Sharma et al. 2022). Although GAN‐generated data may contribute to such frameworks, synthetic data generation alone does not constitute a Digital Twin. A livestock Digital Twin would require longitudinal integration of individual‐level genomic, physiological, environmental, and management information, with GANs potentially serving as one component for modeling plausible biological states.

Hazra et al. (2022) further demonstrated the use of GAN models to generate synthetic feline genomes associated with various disease phenotypes. The generated samples achieved a 93.7% correlation with real genomic data, highlighting the capacity of GAN models to produce genomic profiles that reproduce characteristics of the observed data distribution. Additionally, Ghahramani et al. (2018) successfully applied GANs to single‐cell data and predicted perturbations in these cells over time. In livestock systems, analogous approaches could be developed using omics datasets collected under clearly defined biological conditions. For example, transcriptomic profiles from animals maintained under thermoneutral conditions and exposed to heat stress could be used to train a conditional GAN in which environmental condition is supplied as a model input. Once trained, the generator could produce synthetic transcriptomic profiles that reproduce patterns associated with each condition. The same framework could be applied to nutritional treatments, disease states, reproductive stages, pharmaceutical interventions, or other experimentally characterized conditions.

These synthetic datasets can provide controlled data for methodological evaluation, sensitivity analyses, and exploratory modeling, particularly when the original datasets are small or costly to generate. However, the biological relationships represented in the generated samples remain constrained by the information contained in the training data. Consequently, synthetic observations should not be treated as additional independent animals in analyses intended to estimate treatment effects, identify novel associations, or establish biological mechanisms. Validation should therefore focus on whether the generated observations preserve the biological properties required for the intended application. For transcriptomic data, this could include comparisons of expression distributions, gene–gene correlations, pathway enrichment, and condition‐specific expression patterns between synthetic and real samples. For metabolomic or proteomic data, analogous evaluation could assess metabolite or protein distributions, covariance structures, and known pathway responses. The ability to generate targeted and context‐specific omics data in silico could support methodological development, hypothesis generation, and exploratory research in livestock genetics, functional genomics, and precision breeding.

### 5.3 Addressing Data Imbalance in Livestock Omics

Data imbalance is a common challenge in genomics, particularly when analyzing rare variants, disease‐associated alleles, underrepresented breeds, rare phenotypes, or biological states represented by relatively few animals. GAN‐based architectures, such as Offspring‐GAN and AC‐GAN, previously discussed in the Section 4.2, have demonstrated effectiveness in synthesizing underrepresented classes and balancing datasets (Das and Shi 2022). For instance, Offspring‐GAN has been successfully applied to address ethnic underrepresentation in human genomic datasets (Das and Shi 2022), and similar strategies could be translated to livestock populations to investigate how predictive and classification methods perform when particular groups are poorly represented. A potential livestock workflow would first separate the original data into training and independent testing sets before any synthetic samples are generated. A conditional GAN could then be trained using only the training data, with phenotype, breed, disease status, or another relevant class supplied as the conditioning variable. Synthetic observations could subsequently be generated for the minority class and incorporated into the training dataset, while model performance would be evaluated exclusively using unseen real animals. This design is necessary to determine whether GAN‐based balancing improves model behavior on authentic observations rather than simply improving performance on data derived from the same training distribution.

Cusworth et al. (2024) employed a WGAN‐GP architecture to correct class imbalance in small but complex omics datasets in humans. Their model improved classification performance in several datasets, although its advantage over traditional techniques, such as synthetic minority oversampling and random oversampling, varied according to the dataset and experimental setting. These results indicate that GAN‐based augmentation should be compared directly with simpler balancing strategies rather than assumed to provide superior performance. These methods could be further adapted to livestock omics datasets in which class imbalance affects the development or evaluation of analytical models. Importantly, generating additional synthetic observations from a minority class does not expand the biological variation beyond what is represented in the original samples. If only a small number of animals represent a particular phenotype, breed, or genotype class, the GAN can only learn and reproduce patterns contained within those animals. Therefore, synthetic augmentation cannot substitute for collecting additional real animals when the objective is to estimate genetic effects, identify biomarkers, perform association analyses, or characterize biological variation that is poorly represented in the original population.

Beyond classification, GAN‐based balancing could nevertheless provide a useful framework for evaluating the sensitivity of analytical methods to different levels of imbalance or minority‐class representation. The generated data should also be evaluated for biological fidelity. For genomic data, this could include allele‐frequency distributions, linkage disequilibrium, haplotype structure, and population relationships. For transcriptomic or other functional omics data, synthetic observations should preserve covariance patterns and biological pathways characteristic of the minority class. Such evaluations determine whether the GAN adequately reproduces the available biological variation rather than whether it generates new biological information.

### 5.4 Enhancing the Scalability and Interpretability of GAN

One of the persistent concerns with GANs is their computational burden when training on high‐dimensional genomic data, as well as the interpretability of deep learning models. Szatkownik et al. (2023) addressed this by applying principal component analysis for dimensionality reduction prior to GAN training. The generated data were then inverse‐transformed back into the original space, enabling large‐scale genomic simulation with reduced computational cost. This approach could be particularly useful for livestock breeds or species where whole‐genome SNP datasets can include millions of markers, making GAN training computationally demanding. In livestock applications, a similar workflow could first apply PCA to a real SNP genotype training set and retain a predefined number of principal components representing the major axes of genomic variation. The GAN could then be trained on these component scores rather than directly on the complete genotype matrix. Synthetic profiles generated in the reduced space could subsequently be transformed back into the original marker space and compared with real animals for allele‐frequency distributions, population structure, linkage disequilibrium, haplotype patterns, and other genomic properties relevant to the intended application. However, dimensionality reduction introduces a trade‐off between computational efficiency and preservation of biological information. Components explaining relatively little overall variation may still contain important genomic signals, including rare variants, localized linkage disequilibrium patterns, or variation associated with small subpopulations. Therefore, the number of retained components should be evaluated not only according to computational efficiency but also according to the genomic characteristics that must be preserved in the generated data.

To enhance the interpretability of GAN outcomes, Riley et al. (2024) proposed correlating the discriminator's hidden layers with data summary statistics to improve biological interpretability. This approach relates internal model representations to established population‐genetic statistics, allowing researchers to investigate which genomic properties contribute to the discriminator's predictions. This approach helps mitigate the “black box” criticism often directed at GAN models by providing insight into which features drive the discriminator's decisions. In livestock applications, discriminator activations could similarly be compared with biologically interpretable features such as allele‐frequency patterns, linkage disequilibrium, heterozygosity, haplotype diversity, or functional genomic annotations. For example, if a GAN‐based model were used to identify candidate regions under selection, these analyses could help determine whether the discriminator was responding to plausible population‐genetic signals rather than technical artifacts. Such insight could facilitate model tuning and ensure that generated outputs are biologically plausible, increasing confidence in the downstream application of GAN‐derived data in livestock genomics. Nevertheless, interpretability should be evaluated together with biological validation because a model may reproduce the overall distribution of a dataset while failing to preserve the specific genomic features required for a particular application.

## 6 Concluding Remarks

Generative artificial intelligence is an emerging, rapidly evolving field in computer science, with applications spanning multiple disciplines because it can synthesize diverse forms of data. In particular, the Generative Adversarial Networks (GANs) approach has gained attention for its ability to produce highly realistic outputs, including images, sequences, text, and biological data, without duplicating exact examples from the training set. There is growing evidence supporting the use of GANs in biological research, particularly for generating in silico omics data to enhance the performance of statistical and machine learning models, facilitate privacy‐preserving data sharing, and expand access to sensitive datasets. GANs have shown potential for refining evolutionary models and detecting selection signatures such as positive and balancing selection. In such frameworks, a generator can be trained to simulate sequences under a given demographic model, while a discriminator is optimized to recognize genomic patterns under selection, enabling the discovery of selective processes in real populations. This methodology could be valuable for exploring evolutionary dynamics in livestock species and testing alternative demographic scenarios. Furthermore, GANs offer practical advantages for addressing data imbalance and improving representation in genomic, transcriptomic, and single‐cell datasets, especially for rare variants or traits with low heritability. Notably, GAN models can also be applied to generate de novo biological sequences, improve variant calling and genotype imputation, enhance genome‐wide association studies, predict the functional impact of mutations, and simulate cellular responses to perturbations in silico. In the context of livestock, these capabilities can be leveraged to generate synthetic datasets that augment underrepresented traits or genotypes, identify biomarkers (e.g., miRNAs), and enhance downstream analyses in genomic research pipelines. Looking ahead, extending these approaches to integrated multi‐omics frameworks and leveraging emerging generative paradigms such as diffusion models may further improve biological fidelity and predictive performance in livestock applications. At the same time, challenges related to model stability, evaluation metrics, and interpretability need to be addressed to ensure that GAN‐derived outputs remain robust and biologically credible across diverse populations and environments. This review highlights the growing potential of GANs as a complementary tool in livestock genomics research, offering opportunities to generate synthetic data reflective of biological processes, address key limitations in current datasets, and support the development of more efficient, data‐driven breeding strategies and research studies.

## Conflicts of Interest

The authors declare no conflicts of interest.

## Data Availability

Data sharing not applicable to this article as no datasets were generated or analyzed during the current study.
